# Low Utilization of Insecticide-Treated Bed Net among Pregnant Women in the Middle Belt of Ghana

**DOI:** 10.1155/2017/7481210

**Published:** 2017-07-30

**Authors:** Grace Manu, Ellen Abrafi Boamah-Kaali, Lawrence Gyabaa Febir, Emmanuel Ayipah, Seth Owusu-Agyei, Kwaku Poku Asante

**Affiliations:** Kintampo Health Research Centre, P.O. Box 200, Kintampo, Ghana

## Abstract

**Background:**

Malaria in pregnancy leads to low birth weight, premature birth, anaemia, and maternal and neonatal mortality. Use of insecticide-treated nets (ITNs) during pregnancy is one of the proven interventions to reduce the malaria burden. However, Ghana has not achieved its target for ITN use among pregnant women.

**Methods:**

A qualitative study was conducted in seven communities purposively selected from the middle belt of Ghana. Participants who had delivered in the six months prior to this study were selected. In all, seven focus group discussions and twenty-four in-depth interviews were conducted between June and August 2010.

**Results:**

Respondents knew of the importance of ITNs and other malaria-preventive strategies. Factors such as financial access and missed opportunities of free distribution denied some pregnant women the opportunity to own or use an ITN. Reasons for not using ITNs during pregnancy included discomfort resulting from heat, smell of the net, and difficulty in hanging the net. Participants maintained their ITNs by preventing holes in the nets, retreatment, and infrequent washing.

**Conclusion:**

Pregnant women know about the causes and prevention of malaria. However, this knowledge is not transformed into practice due to lack of access to ITNs and sleeping discomforts among other logistical constraints.

## 1. Introduction

*Plasmodium falciparum *malaria is a major contributor to maternal and neonatal mortality in sub-Saharan Africa [1]. It is responsible for about 25% of all maternal deaths in malaria-endemic areas [2]. Malaria in pregnancy (MiP) is a public health problem [3–5]. In Africa, about 10,000 women and 11% of neonates are estimated to die each year due to malaria infection during pregnancy [1]. According to the Ghana Multiple Indicator Cluster Survey (MICS) conducted in 2011, 3.5 million cases of clinical malaria are estimated to be reported each year in public health facilities, accounting for 38% of outpatient attendance and 35% of all admissions and 34% of deaths in children below five years of age [6]. The main effects of MiP include low birth weight, premature birth, anaemia, and mortality of the newborns [1, 7–9].

Globally, there have been gains in malaria investments over the years and they have resulted in availability of cost-effective interventions and substantial reductions in malaria deaths [5, 10]. However, effective utilization of these interventions to attain the key targets of the National Malaria Control Strategic Plan (2008–2015) in Ghana has not been fully achieved. This strategy among other things aimed to increase the number of pregnant women sleeping under treated nets from the 2008 levels of 32.3% to 80% by the year 2015 [11]. Insecticide-treated nets (ITNs) are one of the proven cost-effective components of malaria prevention through vector control approach. Appropriate use of ITN is shown to reduce malaria transmission by about 90% [6]. Use of ITN during pregnancy is shown to reduce miscarriages and stillbirths by 33% [1, 6].

Large quantities of mosquito nets have been distributed in Ghana as part of the country's efforts to improve access to ITN and to achieve universal ITN use coverage [12]. Initially, ITNs were distributed through either a voucher scheme, maternal and child health campaigns by subsidizing the cost, or selling them at the full cost. In 2010, the free mass distribution initiative was introduced to achieve a universal coverage by 2012 [13]. A door-to-door distribution and hang-up exercise were incorporated into the mass distribution campaign to achieve universal ownership and use. These interventions led to an increase in household ownership of ITN from 49% in 2011 to 68% in 2014 [6, 12]. ITN use among pregnant women in Ghana also increased from around 33% in 2011 to 43% in 2014.

Lack of access to ITNs and poor knowledge and perception on ITNs and malaria have been previously reported as important barriers to the use of ITNs in parts of Africa [14]. However, access does not always result in usage due to sociocultural and logistical reasons [15]. Evidence from some parts of Ghana has shown that over 40% of ITNs available in the households go unused [16]. This could erode the gains made in ITN use over the years.

In this study, a qualitative approach was used to assess factors associated with ITN use among pregnant women in the middle belt of Ghana. Issues related to maintenance and disposal practices of ITN among pregnant women in the study area were also examined. In addition, the study assessed other malaria prevention strategies among pregnant women.

## 2. Materials and Methods

### 2.1. Study Area

**Figure 1:**
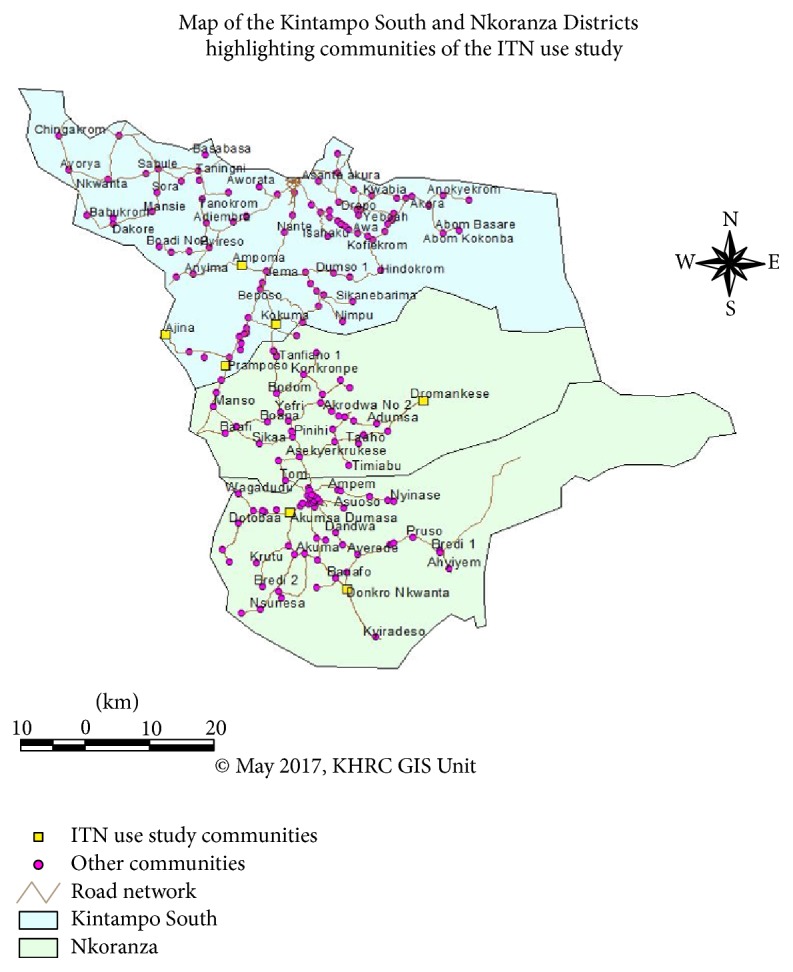


The study was conducted in the Kintampo Municipality and South District and Nkoranza North and South Districts in the Brong-Ahafo region (Figure 1). They are located within the forest-savannah, transitional ecological zone of Ghana. The area is mostly rural with residents mainly being farmers. Residents are of diverse ethnic backgrounds with Akans being the predominant ethnic group. The Akan language (Twi) is widely spoken and understood by almost all residents [17]. The mean monthly temperature is between 18°C and 38°C with rainfall averaging 1250 mm per annum. Malaria parasite prevalence among children below 5 years of age in the study area was about 50% in 2009 and the entomological inoculation rate was about 269 infective bites per person per year [18]. A birth cohort study conducted in 2010 in the study area showed that the prevalence of placental malaria was about 40% [19].

### 2.2. Study Design

This was a qualitative cross-sectional study conducted between June and August 2010. A pretested interview guide was used to explore responses on preidentified themes such as reasons for not using ITNs. Focus group discussions (FGDs) and in-depth interviews (IDIs) guides were the main data collection tools used. All IDIs were conducted in the respondents' homes. FGDs were also held at selected places convenient for the respondents. Two research officers (males) with extensive experience in qualitative methods conducted the interviews. One research officer was responsible for moderating the interviews, while the other research officer took notes of the discussions. Interviews were conducted in the local dialect (Twi) and were audio-recorded with a digital recorder. Observations of ITNs in the communities were also recorded as pictures using a digital camera and notes.

### 2.3. Selection of Study Participants

Study participants were drawn from the Kintampo Birth Cohort Study. In the Kintampo study, pregnant women were enrolled into the study and followed up monthly till delivery. During follow-up visits, they were asked whether they slept under an insecticide-treated net the night before the visit in addition to other questions as described elsewhere [19]. Women who did not sleep under ITNs the night before the visit on two or more occasions were purposively selected for this study. They all delivered between January 2010 and June 2010 and were chosen based on logistics, convenience, and availability. In all, a total of seven FGDs and twenty-four IDIs were conducted in seven selected communities (Table 1). Each FGD included between seven and twelve participants (Table 1).

### 2.4. Data Management and Analysis

Recorded interviews were numbered according to the order of the interviews. Each recorded interview was translated into English and transcribed verbatim. They were then compared with their corresponding notes in order to ensure accuracy. A thematic analysis was done by grouping similar responses under major themes in a matrix form based on the objectives of the study. Illustrative quotes are presented to support the findings. Quotations are identified by the respondents' number.

### 2.5. Ethical Approval

The survey was approved by the Kintampo Health Research Centre's Institutional Ethics Review Committee (Federalwide Assurance number: 00011103). The objectives of the study were explained to them and written informed consent was obtained from all respondents prior to participation. There were no other risks and participants were assured of confidentiality at the consenting stage of the study. Transcribed data are kept confidential on a password-protected computer and are accessible to only named investigators.

## 3. Results

### 3.1. Characteristics of Respondents

The median age of participants was 24 years (with range from 14 to 46 years). Generally, most of the respondents (78.5%) had received some kind of formal education. This ranged from primary to technical or senior high school. About half (52.7%) of them were living with a man (Table 2).

### 3.2. Knowledge about Insecticide-Treated Bed Nets

All respondents knew about insecticide-treated nets (ITNs) and their importance in malaria prevention. They perceived sleeping under ITNs as very beneficial because it protects a pregnant woman and her unborn child from malaria which can lead to other health problems. About 20% of participants also spoke about the sound sleep derived from sleeping under an ITN. This was credited to the ability of ITNs to serve as a barrier to the buzzing noise made by mosquitoes at night.

*…the treated net protects the pregnant women mbecause [sic] if you don't sleep in it and you get mosquito bites, it will infect the unborn child in the womb with malaria.* -(R4: FGD, Pramposo)

### 3.3. Factors Affecting the Use of Insecticide-Treated Bed Nets

The respondents gave different reasons why some pregnant women do not sleep under ITNs every night. These reasons were categorized under four themes: access/mode of acquisition, discomfort, and logistical constraints.

### 3.4. Access/Mode of Acquisition

About 53 (57.2%) of respondents owned ITNs at the time of the interviews and they reported to have had them free of charge through free distribution campaigns in the communities and at health facilities. A few others (7.8%) bought theirs from the open market at full cost. Those who did not have ITNs (35%) attributed that to a missed opportunity of the free distribution exercise, unavailability of ITNs at the health facilities where prices are subsidized, and lack of money to purchase one from other sources. This is captured by the following response:

*ITNs are sold at the health facility at a lower price. If you don't go early you might notget [sic] it to buy because a lot of people go there to get them. If you miss this, then you have to buy it from the open market at a relatively higher price. So if you don't have the money, then you cannot get the net to use.* (IDI, Dromankese)

*As for me, it is because I do not have one (ITN) otherwise, I would have always slept under it. When you have money, you can buy one, but if you do not have money, it means you cannot get it (ITN).* (Pramposo IDI)

### 3.5. Discomfort with Usage

Over 90% of respondents found ITNs to be uncomfortable to use, especially during pregnancy, because they entrap heat during warm weather. This made it uncomfortable for about 15% of pregnant women to sleep under the nets without extra supply of fresh cool air. The chemical in the nets was described to have unpleasant smell that made about 15% of pregnant women vomit or experience difficulty in breathing. Below are some of the comments made in this direction.

*Because of the medicinal scent in the net, some pregnant women do not want to sleep in it, because it will make them vomit though sleeping in an ITN will prevent you from getting malaria.* (R6: FGD, D/Nkwanta)

*There is always heat in the ITNs so during pregnancy it becomes uncomfortable to sleep in. You would therefore wish to pour water on the ground to sleep on. That is why we cannot sleep in an ITN.* (R5: FGD, Akumsa Domase)

### 3.6. Logistical Constraints

**Figure 2:**
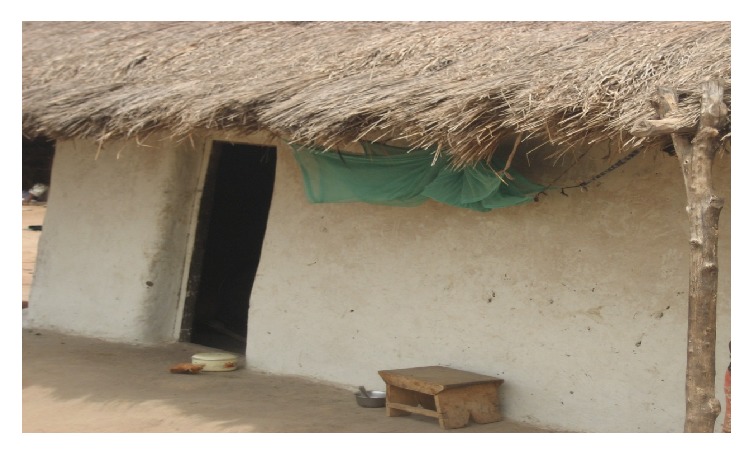
ITN (in green) is hung outside the sleeping room.

About 20% of respondents mentioned that they did not use their nets because it was difficult to hang them. The art of hanging ITNs is a cumbersome task and others (9%) also found it a challenging process due to the architecture of their rooms and beds. In certain rooms, there were no structures to hang the ITNs on and where they were able to hang them, ITNs frequently got dirty because they are hung over bed linins on the bare floor. Majority (63%) of the respondents slept outside of their rooms especially when the weather was hot, which helped them find alternative means of hanging the nets outside (Figure 2).

The size of ITNs was also mentioned as a barrier to ITN use by some (14.2%) respondents. Some respondents did not sleep in their ITNs because the nets are not big enough to accommodate themselves and other family members who share the bed with them. It was a common practice in the communities for women to share one sleeping material with other family members, especially their children.

*I share a common sleeping material with my children and we all cannot sleep under one ITN that is why I do not enjoy using an ITN.* (IDI, Ampoma)

### 3.7. Maintenance of ITNs

Respondents maintain their ITNs by preventing of holes in the nets, infrequent washing, reimpregnation, and storing them properly. Respondents prevented holes in their ITNs by folding them every morning so that children do not play with them. Other measures employed to avoid holes included fixing the nets firmly against strong structures to avoid them falling off every time, reducing tension at the sides of the nets and avoiding overcrowding in the net and reducing excessive movements in the nets when sleeping. They also keep their ITNs in a cool place to avoid a decay of the insecticide in the nets. These views are indicated in the following response:

*You can fold it at the top in the morning due to the children, who might play with it and reset it in the evening to prevent the children from tearing the nets.* (IDI, Kokuma)

### 3.8. ITN Disposal Practices

Almost all respondents (97%) dispose of their nets at the refuse dump; the remaining few (3%) bury them in the pit, burn them, or use them as a household material such as a rag or toilet tissues. Others, however, keep them as an evidence of having used an ITN.

*You can throw it at a refuse dump, or you can use it as rags to carry things or tear it as a tissue for toilets or you may fold it and hide it somewhere.* (IDI, Akumsa Domase)

*The community health nurses complain that we don't sleep in the net, so I don't throw it away. I keep it to serve as an evidence when we are confronted with questions about not using the net and for that matter, falling sick.* (R8: FGD, Ampoma)

### 3.9. Other Malaria Prevention Strategies

Apart from ITNs, respondents also mentioned some vector control measures of preventing malaria. These have been categorized into environmental measures that destroy breeding grounds of mosquitoes and other strategies that serve as a barrier between man and the mosquito.

### 3.10. Environmental Prevention Strategies

In all interview sessions, respondents indicated that strategies for environmental malaria control could include weeding of surroundings, distilling gutters in the vicinity, and removing polythene bags and emptying containers from their premises. These measures were thought to be efficient in preventing mosquitoes and hence malaria. Some participants had the following to say:

*What I will say is that we should get rid of things like containers that can hold water from the environment because these things serve as breeding grounds for mosquitoes.* (R10: FGD, Ampoma)

### 3.11. Other Strategies

Some other methods used by respondents as barriers to mosquito bites included wearing of protective clothes (long dresses), as indicated by almost all respondents (98%), use of electric fans (28%), sealing of eaves around rooms (14%), use of window nets (28%), use of trap doors (28%), and ensuring closing doors and shutting windows at all times (76%).

*It is because of malaria, that is why we are all talking about mosquitoes, so when you are at home, you need to put on long sleeved clothing, as well as trousers, so that mosquitoes cannot bite you to make you get malaria.* (R6: FGD, Dromankese)

*You have to use trap doors and nets to prevent mosquitoes from entering the room. You can also use fans in your room to blow the mosquitoes away.* (R10: FGD, D/Nkwanta)

About 51.7% of respondents mentioned the use of mosquito coil, mosquito spray/indoor residual spray, and repellants or burning other substances to drive mosquitoes away to prevent their bites.

*You can buy mosquito coil and light it in your room to prevent the mosquitoes. You can also burn the palm kennel to prevent mosquitoes in our homes.* (IDI, Kokuma)

### 3.12. Community Bye-Laws for Malaria Control

There were bye-laws that enjoin household members to properly dispose of these waste water receptacles as a way of preventing mosquitoes from breeding. The laws also ensure that choked gutters and bushy environments around houses are cleared by household members.

There were punitive sanctions for persons who fail to comply with these directives.

Respondents appropriately identified the community leadership and sanitary inspectors as the agents responsible for enforcing the bye-laws and punishing members who flout the laws.

*In this community, when you fail to comply with these laws and you are found guilty, you will be charged a fine 10 Ghana Cedis [equivalent to five US dollars at the time of the survey].* (IDI, Dromankese)

*The sanitary inspectors walk around, when you do not obey such laws, they will call you for verbal warning and later follow it up with summon to the court.* (IDI, Pramposo)

## 4. Discussion

The objective of this study was to assess factors associated with ITN use among pregnant women in the middle belt of Ghana. The study also assessed other malaria prevention strategies among pregnant women. Issues related to maintenance and disposal practices of ITN among pregnant women in the study area were examined as well.

This study showed that the use of ITNs as a malaria prevention measure was well known by respondents. However, as reported from the Kintampo Birth Cohort Study conducted in 2010, ITN use among women in the study area was low, 47% [19]. In a previous study conducted in Nigeria in 2012 [20], knowledge about the usefulness of ITNs in preventing malaria and the risk of malaria in pregnancy was concluded as the most likely factor required to increase ITN use. This does not seem to be the same in Ghana.

Factors such as lack of access to ITNs, logistical constraints, and discomfort associated with the use of the ITNs were cited as the reasons for not using ITNs in this study. ITN ownership has been a common factor for not using ITNs in other studies [14, 15], though ownership in itself does not guarantee the use of ITNs [21]. In this study conducted in 2010, most of the respondents (57.2%) had received free ITNs or had purchased ITNs at subsidized rates from a public health facility. Some participants missed such opportunities and needed extra income to buy ITNs at full cost that was relatively higher if they intended to use them. However, during the second half of 2010, there was a free door-to-door distribution and hang-up of ITNs in the country [13] that provided a further opportunity for pregnant women to own an ITN.

Free delivery of ITNs has been shown not to necessarily increase ITN use [22]. Some studies have shown that a personal decision to purchase a net may motivate one to use the ITNs [16] rather than getting it for free. Therefore it is important to encourage commercial distribution of ITNs but at a subsidized price that is at the barest minimum [23]. Such a strategy, backed by education on the proper care and maintenance of the nets to ensure their durability, is also likely to lay the foundation for a sustained supply of ITNs.

While about 20% of respondents reiterated the pleasure derived from sleeping under ITNs given that they do not hear the buzzing noise made by mosquitoes as in other studies [24, 25], another 40% of respondents detest sleeping under treated bed nets. The respondents stated reasons such as discomfort due to heat from the net and difficulty of hanging the nets as challenges in using ITNs. These observations are similar to findings in other studies in Africa [14, 20, 26–29]. More aerated materials other than polyester may help to enhance ventilation through hung ITNs. The use of other preventive tools such as cream repellents should be encouraged especially during the hot seasons when pregnant women are unlikely to use ITNs.

The strong chemical scent in the ITN was cited as a strong disincentive for using ITNs by about 55% of respondents. It also could cause body itching and vomiting. This effect may be curbed by educating people to hang the net in an airy place for at least 24 hours before use [13]. As health education is an influential factor in malaria control, future mass distribution exercises should consider incorporating this information in the programmes' educational messages [30]. Other retailers should make this information available to clients as a way of managing ITN user expectations.

Maintenance is an important element in sustaining ITN coverage over time and in preventing malaria infections. Worn-out nets have been a barrier to the use of nets in some studies [28]. All participants in this study could avoid holes in their nets by observing some simple practices such as folding them up every morning. Only few respondents mentioned reimpregnation of their ITNs because the practice is likely to be fading away as newer brands of ITNs are long-lasting and can last for about 20 washes before the insecticide fades out [13].

Apart from using ITNs, all respondents acknowledged the use of other malaria-preventive strategies such as the use of mosquito repellents, wearing protective clothes, and practice of environmental hygiene as described in Results as a way of controlling malaria similar to other findings [25]. ITN disposal practices were mixed and were inconsistent with standard disposal practices. Respondents mentioned practices such as burning and discarding ITNs in a pit or at the refuse dump or use of ITNs for other purposes such as rags and toilet tissue. This observation is similar to that of the study of Timor-Leste, in which nets were observed to have been used for other purposes such as fishing nets and also to protect backyard gardens [25]. In this study, there was no mention of a formal collection and disposal of used ITNs by health professionals for disposal as per the WHO guidelines [31].

## 5. Limitations

Participants in this study were women who had delivered in the past six months (between January and June 2010) and they responded to questions on ITN use during pregnancy. Therefore, recall bias might have affected their responses. Due to limited resources, communities that had larger numbers of potential respondents were conveniently selected for the study and therefore the results may have limited generalizability.

## 6. Conclusions

Pregnant women know the benefits of ITNs but may not use them for reasons such as lack of access to ITNs, sleeping discomforts when ITNs are used, and difficulty in hanging the nets among other logistical constraints. There is therefore an urgent need for all stakeholders to intensify behavior change communication interventions to facilitate the adoption of ITN use by pregnant women. Beyond the use of ITNs, other means of protecting pregnant women from malaria must be explored.

## Figures and Tables

**Table 1:** Study communities with number of respondents for FGDs and IDIs (June–August 2010).

Community	Number of participants	
	FGDs	IDIs
Ajena	12	2
Akumsa Domase	12	4
Ampoma	8	4
Donkro Nkwanta	11	3
Dromankese	11	4
Kokuma	7	3
Pramposo	8	4

**Table 2:** Demographic characteristics of both FGD and IDI respondents (*n* = 93).

	Number of respondents	Percentage
*Age groups*		
14–18	12	12.9
19–24	35	37.6
25–34	39	41.9
35–49	7	7.5
*Educational level*		
No education	20	21.5
Primary school	32	34.4
Middle school, JSS/JHS	36	38.7
Post-middle college (e.g., training/secretarial)	2	2.2
Technical/commercial/SSS/SHS	3	3.2
*Marital status*		
Single	4	4.3
Living together	49	52.7
Married	36	38.7
Separated	4	4.3
*Type of occupation*		
Not working	16	17.2
Labourer/domestic worker/farmer	35	37.6
Professional teacher/nurse/accounts clerk	3	3.2
Seamstress/hairdresser	9	9.7
Trader/food seller	30	32.3
*Ethnicity*		
Akan	55	59.1
Bimoba, Chokosi	1	1.1
Dargarti	20	21.5
Fulani	1	1.1
Ga, Adangbe, Ewe	1	1.1
Gonja, Dagomba, Mamprusi	2	2.2
Hausa	1	1.1
Konkomba, Basare	4	4.3
Mossi	1	1.1
Sisala, Wala	7	7.5
